# Does learning more about others impact liking them? Replication and extension Registered Report of Norton *et al*.’s (2007) lure of ambiguity

**DOI:** 10.1098/rsos.250441

**Published:** 2025-04-30

**Authors:** Zöe Horsham, Ashleigh Nicola Haydock-Symonds, Hirotaka Imada, Hiu Ching Tai, Wing Lam Lau, Tsz Lui Shum, Yuqing Zeng, Hiu Tung Kristy Chow, Gilad Feldman

**Affiliations:** ^1^ University of Kent, Canterbury, England, UK; ^2^ Royal Holloway University of London, Egham, England, UK; ^3^ The University of Hong Kong, Hong Kong; ^4^ Psychology, The University of Hong Kong, Hong Kong

**Keywords:** impression formation, liking, less is more, similarity, ambiguity, curiosity, registered report, replication, decision-making

## Abstract

Norton *et al*., 2007, demonstrated a counterintuitive phenomenon that knowing other people better and/or having more information about them is associated with decreased liking. They summarized it as ambiguity leads to liking, whereas familiarity can breed contempt. In a Registered Report with a US Prolific undergraduate student sample (*N* = 801)*,* we directly replicated Studies 1a, 1b and 2 and conceptually replicated Studies 3 and 4 from Norton *et al*., 2007. Extending their research, we also proposed that curiosity provides an alternative path to liking, hypothesizing that curiosity mediates the relationship between knowledge and liking. Overall, we found weak support for the original findings. Consistent with the original article, participants believed they would like someone who they knew more about (original: *h* = 0.52–0.70; replication: *h* = 0.55–0.75) and that knowledge positively predicts liking (original: *h* = 0.21–0.45; replication: *h* = 0.57–0.76). However, we found no indication of the number of traits known influencing liking (original: *r =* −0.43 to −0.005; replication: *r* = −0.05 to 0.06) or perceived similarity to the target (*d* = 0.00), for a mediating effect of perceived similarity, for a dissimilarity cascade effect, or for changes in liking or perceived similarity as a factor of learning more about the target. In our extensions, we found support for a positive relationship between curiosity and liking (*r* = 0.62–0.70), but not for knowledge and curiosity (*r* = −0.06 to 0.05). Overall, our findings suggest that learning more about others may not influence perceptions of liking, similarity or curiosity towards them. Materials, data and code are available on https://osf.io/j6tqr/. This Registered Report has been officially endorsed by Peer Community in Registered Reports: https://doi.org/10.24072/pci.rr.100947.

## 1. Introduction

Initial encounters are abundant in our social lives, and multiple encounters with the same acquaintance^1^ are regular occurrences ([1]; see also [2]). People often wish to keep and build a relationship with some acquaintances, actively seeking to meet them to establish friendships and romantic relationships. However, when meeting someone for the first time, there is virtually no information about an individual. How do people form and develop initial impressions of others, and how does our knowledge of them influence our liking towards them? We argue it is imperative to accurately understand how people form social perceptions and evaluations of new individuals as they acquire more information about them, thus helping us predict and elucidate how people develop new relationships.

Addressing this question, Norton *et al*. [3] stipulated that a lack of information about others, which forms ambiguous positive expectations, increases the perceived attractiveness of others, a.k.a., the *lure of ambiguity* effect. Furthermore, they asserted that these overly positive initial impressions decrease as people begin to know more about others, since this reveals dissimilarities rather than similarities. They coined this notion the *less is more* effect. Intuitively, though, it is tempting to assume that we like others more as we know more about them, and there are studies suggesting so; familiarity is an essential component in the formation of both romantic and non-romantic relationships ([4–6]; see also [7]). Reis *et al*. [5] also claimed that individuals actively highlight commonalities with others to promote an engaging conversation, thus leading to favourable impressions and subsequent attraction. Given the degree of homophily identified among society ([8]; see also [9]), more information appears to lead to more liking as a result of previously unknown similarities that are accentuated during initial encounters. Nevertheless, supporting the *less is more* effect, Norton *et al*. [3] demonstrated that people’s liking of others is greater when they know less about them. The authors argued—and provided further support in a later publication [10]—that the *less is more* effect can be observed within everyday life, such as the cessation of friendships, business relationships, and marriages.

Norton *et al*. [3] highlighted the lure of ambiguity during impression formation. The authors drew on the *person positivity bias* during initial encounters, in that people tend to view strangers positively when there is little information available about them ([11]; see also [12]). Specifically, they suggested that ambiguous targets are initially perceived as being more similar [13–15], with skewed assumptions that others may share some features with them such as personality traits (i.e. a *false consensus effect*; [16]). This misperception of similarity may result in the initial liking of the target (see [17,18]). Once information about the ambiguous target is revealed, this overly positive state and overestimation of similarity wane, and correspondingly, liking is reduced. In other words, the authors suggested that perceived similarity mediates the relationship between knowledge and liking. Furthermore, they proposed that when erroneous assumptions of similarity are met with unexpected evidence of dissimilarity, subsequent information is interpreted as compounding evidence of further dissimilarity.

### 1.1. Target for replication: Norton *et al*. (2007)

We chose Norton *et al*. [3] for a direct replication for three reasons: its impact, mixed findings in the literature and the lack of direct replications. Norton *et al*. [3] conducted a series of studies examining whether manipulating the amount of information presented about others impacts perceived liking and showed that those presented with higher numbers of pieces of information regarding others’ traits tended to report less liking towards them (i.e. the *less is more* effect).

First, the article has had an impact on the literature, with 362 Google Scholar citations at the time of writing (July 2023). Second, previous studies collated mixed evidence as to how knowledge influences liking. Norton *et al*.’s [3] findings, in fact, are inconsistent with well-established literature suggesting the opposite, which led to a debate between Reis *et al*. [5] and Norton *et al*. [19]. Reis and colleagues discussed the nature of the relationship between familiarity and/or information and liking, using different paradigms. This led to an attempt by authors on both sides to integrate the findings into one unified paradigm [20], though this paradigm still requires further empirical testing.

Ullrich *et al*. [21] also challenged the findings by Norton *et al*. [3]. Using the same materials and between-participants design as Norton *et al*. [3], they found no support for more information being associated with decreased liking. There were, however, minor modifications to Norton *et al*.’s [3] methodological approach, such as the use of a single-item measure of self-esteem and changes to the wording of instructions, that diminish the ability to directly compare their findings to the target article. To review or resolve these disagreements is beyond our intended scope, yet we consider it a necessary first step to revisit the findings to ensure they are reliable, consistent and generalizable.

To the best of our knowledge, there are currently no published direct pre-registered independent replications of the target article nor any of the follow-up articles, which raises the need for a direct replication registered report employing open science best practices with high statistical power.

### 1.2. Replication: Norton *et al*. (2007): Studies 1a/b, 2, 3 and 4

We conducted a direct replication of Norton *et al*.’s [3] Studies 1a, 1b and 2, and we conceptually replicated Studies 3 and 4.^2^ We did not include Studies 3 and 5 as targets of direct replications, as these involved experiments using real online dating platforms. We summarized our replication hypotheses and target effect sizes for replication in Table 1.

**Table 1.** Summary of hypotheses and effect sizes of the target article and replication.

study	operationalized hypothesis	replication	effect type	target article’s effects		replication effect	
				effect size [95% CI]	conclusion	effect size [95% CI]	conclusion
1a	H1a: individuals prefer a person who they know more about compared to a person they know less about	direct	*h*	0.62 [0.52, 0.70]	H1a supported	0.65 [0.55, 0.75]	signal—consistent (replication CI does not include 0 and includes the original ES)
1b	H1b: individuals believe that more information leads to more liking rather than less liking	direct	*h*	0.34 [0.21, 0.45]	H1b supported	0.67 [0.57, 0.76]	signal—inconsistent larger (replication CI does not include 0 but excludes the original ES)
2	H2-1: the number of pieces of information someone knows about a person negatively correlates with the degree of liking towards them	Direct	*r*	−0.23 [−0.43, −0.005]	H2-1 supported	0.01 [−0.05, 0.06]	no signal—inconsistent (replication CI includes 0 and excludes the original ES)
2	H2-2: the more pieces of information about a person someone receives, the less they are to like the person	conceptual	Cohen’s *d*	N/A	N/A	0.001	H2-2 not supported: No consistent evidence for less liking towards a target with more known information
2	H3: perceived similarity mediates the relationship between the number of pieces of information about a person and liking towards them	conceptual (exploratory)	*r*	−0.006	H3 supported	−0.00001 [−0.001, 0.001]	no signal—inconsistent (replication CI includes 0 and excludes the original ES)
4	H4-1: those presented with initial evidence of dissimilarity to the target perceive subsequent attributes as more dissimilar to themselves than those presented with initial evidence of similarity to the target	conceptual	Cohen’s *d*	0.66 [0.37, 0.95]	H4-1 supported	0.14 [−0.03, 0.30n]	no signal—inconsistent weaker (replication CI includes 0 and excludes the original ES)
4	H4-2: those presented with initial evidence of dissimilarity to the target like the target less than those presented with initial evidence of similarity to the target	conceptual	Cohen’s *d*	N/A	N/A	0.44 [0.28, 0.61]	H4-2 supported: those under initial impressions of dissimilarity showed lower liking towards the target after all traits presented
	H5: the number of pieces of information someone knows about a person negatively correlates with curiosity towards them	Extension	*r*	N/A	N/A	0.005 [−0.06, 0.05]	H5 not supported: no evidence for a relationship between the number of pieces of information known about the target and curiosity towards them
	H6: curiosity is positively correlated with degree of liking	extension	*r*	N/A	N/A	0.66 [0.62, 0.70]	H6 supported: higher levels of curiosity associated with greater liking
	H7: curiosity has an indirect effect on the relationship between the number of traits known about a person and degree of liking towards them	extension		N/A	N/A	N/A	N/A
	H8: people in the dissimilar condition (i.e. those who perceive the first presented trait as evidence of dissimilarity) like the target less as they receive more pieces of information about them	Extension	*Regression beta*	N/A	N/A	−0.05 [−0.15, 0.06]; 0.02 [−0.09, 0.12]	H8 not supported: no change in liking towards the target for those in the dissimilar condition as they receive more pieces of information about them
	H9: people perceive the target to be more and more dissimilar to them as they receive more pieces of information about the person	Extension	*Regression beta*	N/A	N/A	0.05 [−0.05, 0.15]; −0.08 [−0.19, 0.02]	H9 not supported: no change in similarity perceptions towards the target as they receive more pieces of information about them

Further details on the evaluation criteria using [22] are provided in the electronic supplementary material. H2-2 aids H2-1 with experimental approaches and serves as a conceptual replication. As such, there are no original effect sizes for this hypothesis. Confidence intervals were not computed for H2-2. Based on the reported numbers and statistics, we could not compute the confidence interval for the effect size for H3. Effect size for H7 is not reported due to mediation analysis not being conducted, given that H5 was not supported. Effect sizes for H9 refer to perceived similarity when five traits have been presented and when 10 traits have been presented, respectively.

#### 1.2.1. Direct replications of Studies 1a, 1b and 2

In Studies 1a and 1b, Norton *et al*. [3] found that whether presented with a set number of traits (e.g. 1 versus 2 traits or 3 versus 6 traits) or a hypothetical scenario, student participants believed that they would like those they know more about more than those they know less about. More specifically, in Study 1a, participants compared expected liking of targets with different numbers of known traits (e.g. contrasting a person about whom they knew one trait versus a person about whom they knew two traits, etc.). In Study 1b, participants were asked to indicate whether, when meeting an individual for the first time, they tended to like a person more when they know more or less about the person. Across the two studies, they demonstrated that people believe there is a positive relationship between the number of known traits about others and their liking of them. In other words, people intuitively believe that knowing more about others leads to liking them more, in stark contrast with the *less is more* effect. We would like to note that Norton *et al*. [3] claimed that their studies offered evidence for the association between perceived familiarity and liking, as they operationally defined the number of known traits as an index of perceived familiarity. Nevertheless, they did not measure perceived familiarity, and the psychological mechanisms underlying the relationship warrant further elucidation, which we turn to later with our extension.

In Study 2, participants were presented with either 4, 6, 8 or 10 randomly selected traits from the list of 28 traits taken from prior research [23–25] and asked to rate how much they would like an individual with these traits. They thus had a 1 × 4 between-subject design. Despite the experimental design, they treated the manipulated number of presented traits as a continuous variable and computed its correlation with liking, an empirical shortcoming which we aimed to address with our extension (see §4.2. Extensions). They found support for a negative relationship, resulting in the claim that more knowledge led to less liking. While people believe that the more they know about others, the more they like them (Studies 1a and 1b), the level of information they had about a person was in fact associated with lower levels of liking (Study 2). These contrasting results revealed a contradiction between the lay intuition about their liking of new individuals (i.e. more is more) and their actual tendencies (i.e., less is more).

It is worth noting that the discrepancies in experimental design between Studies 1 and 2 may bear some part in the findings. Study 1 used a within-participants design based on a mere comparison of the number of unspecified traits a person possesses, whereas Study 2 used a between-participants design, which involved rating liking towards a series of specific traits in turn. It may be that when traits are not specified, individuals assume these unspecified traits are ones they themselves possess. This may explain why individuals showed greater preference towards a person with a higher number of traits in Study 1; if they assumed these traits were ones they themselves possessed, a higher number of similar traits may lead to more liking. Furthermore, the comparative nature of Study 1 means the linearity of the relationship between the number of traits and liking towards the target is difficult to establish. It is possible that there is a diminishing or even curvilinear relationship between these factors. Alongside the direct replication of the correlation, we carried out a 1 × 4 ANOVA and post hoc pairwise comparisons to extend Norton *et al*.’s [3] methods, aiming to more accurately test their claim and go beyond their correlational result (see Table 1).

#### 1.2.2. Conceptual replications of Studies 3 and 4

In Study 3, Norton *et al*. [3] examined the mediating effect of perceived similarity on the relationship between the number of pieces of information people had about another person and the liking of the person. Study 3 meant to replicate the effect found in Study 2, using a more ecologically valid series of self-generated traits. We chose not to conduct a direct replication of Study 3 because (i) the article did not specify the list of traits, and (ii) Study 3 had a similar design and methodology to that of Study 2, and with our unified design, having both studies run together would be too repetitive. To address the added contributions of Study 3, we instead added the measure of perceived similarity from Study 3 to our replication of Study 2 and tested the mediation in that design. As such, our replication of Study 2 served as both a direct replication of Study 2 and a conceptual replication of Study 3, measuring against a slightly smaller range of traits presented to the original Study 3.

In Study 4, Norton *et al*. [3] tested a cascading effect of dissimilarity that was argued to be responsible for the emergence of the *less is more* effect. They argued that a cascade exists during impression formation, where one instance of dissimilarity causes subsequent information about others to be interpreted as further evidence of dissimilarity. Using 10 random traits taken from Study 2, student participants were asked whether each trait was one they would use to describe themselves. Norton *et al*. [3] found that participants saw the second to tenth presented traits as instances of dissimilarity more often when the first presented trait was one that did not describe themselves, compared to when it was one that did. They thus treated the study as a quasi-experimental design by categorizing participants into one of the two groups, based on whether they found the first presented trait to be similar or dissimilar to themselves. Norton and colleagues then computed a correlation between the number of traits that participants found to be similar to themselves and liking of the target (a binary variable: yes/no to the question of whether they liked the person). They found a positive correlation between the number of traits participants rated as similar to themselves and perceived liking. The authors concluded that the first instance of dissimilarity is associated with less liking because this leads people to see newly obtained information about others as further evidence of dissimilarity (i.e. the cascading effect), and this increase in perceived dissimilarity leads to less liking. However, we found the choice of analytic strategies somewhat arbitrary; to directly test the effect of the quasi-experimental condition on liking, it is sensible to conduct a *t*‐test rather than computing the correlation. We computed this correlation for the purpose of replication and included this analysis in the electronic supplementary material. Our primary analysis, included in the main manuscript, was a *t*‐test to assess whether the quasi-experimental condition influenced liking.

We would also like to note that their Study 4 did not in fact allow us to observe and test the cascading nature of dissimilarity, as they did not measure perceived dissimilarity and track its change overtime. To address this, we introduced questions to measure perceived (dis)similarity and liking when participants were presented with the first, the fifth and the tenth (last) traits, such that we could directly demonstrate the cascading effect. Given these new stimuli, our replication of Study 4 was conceptual rather than direct (see §2.9.5. Replication closeness evaluation).

### 1.3. Extension: Curiosity about the target

Within the replication of Study 2, we introduced an additional variable as an extension: curiosity towards the target. Curiosity is broadly defined as the desire for new information [26,27], which past research has identified as a separate construct within the broader category of information-seeking [28–30]. Curiosity can be conceptualized as either a trait or state construct; trait curiosity encapsulates an individual’s innate tendency to experience curiosity, while state curiosity refers to the variability in curiosity experienced during a given context [31,32]. Given the methodological approach to assess immediate liking in response to information presentation during impression formation, we focused on state curiosity in this study.

We chose curiosity, as it relates directly to ambiguity studied in the target article, whereby knowing less information about a given target may result in increased curiosity towards them. Both constructs relate to information gaps occurring in ambiguous scenarios [33,34], in which curiosity is either positively motivated by the anticipation of new information or negatively motivated by the feeling of deprivation from lack of information [35]. Regardless of the motivations underlying curiosity, ambiguous contexts may generate an information-seeking mindset that is associated with heightened levels of curiosity. This curiosity may be associated with levels of liking towards the target [36]. In other words, we anticipated that curiosity would generate an alternative pathway to the *less is more* effect; the more people get to know about others, the less curious they feel about them and, in turn, the less they like them.

### 1.4. Pre-registration and open science

We provided all materials, data and code on https://osf.io/j6tqr/. This Registered Report was submitted to *Royal Society Open Science* following peer review and recommendation for Stage 2 acceptance at the *Peer Community In* (PCI) *Registered Reports*' platform. Full details of the peer review and recommendation of the paper at PCI Registered Reports may be found at the links below. After submission to the journal, the paper received no additional external peer review but was accepted on the basis of the Editor’s recommendation according to the RSOS PCI Registered Reports' policy (https://royalsocietypublishing.org/rsos/registered-reports#PCIRR). Stage 1 recommendation and review history: [37]; https://rr.peercommunityin.org/articles/rec?id=496; https://osf.io/7mc4y/ (our frozen pre-registration version of the entire Stage 1 packet: https://osf.io/cnakg/). Stage 2 recommendation and review history: [38]; https://doi.org/10.24072/pci.rr.100947. All measures, manipulations and exclusions conducted for this investigation are reported, and data collection was completed before conducting the data analyses. The project was part of a large mass replications and extensions project, which received ethics approval from the University of Hong Kong (#EA220438). This Registered Report was written using the Registered Report template by Feldman [39].

## 2. Method

### 2.1. Power and sensitivity analyses

We first computed target effect sizes for direct replication (summarized in Table 1). Effect size and confidence intervals were calculated with R (v. 4.1.2 [40]) with the help of a guide by Jané [41], and power analyses were then conducted with a combination of R and GPower (v. 3.1.9.6 [42]) for the factors that the authors found support for in the target article (flagged as significant results). We conducted a series of *a priori* power analyses based on these effect sizes, and we found that we require 289 participants to detect the effects reported in the target article with 95% statistical power^3^ at α = 0.05 (see electronic supplementary material, table S1, and analysis code for more details).

Given the likelihood that the original effects are overestimated, we used the suggested Simonsohn [43] small telescopes approach with the generalized rule of thumb of multiplying the largest required sample size among all target studies (289) by 2.5 to 723, rounding up to 800 participants. A sensitivity analysis indicated that a sample of 800 would allow the detection of *d* = 0.23 for independent *t*‐test contrasts and *r* = 0.12 (both 95% power, α = 0.05, one-tail), typically considered weak to medium effects in social psychology research [41] and half or less than the effects reported in the target article.

### 2.2. Participants and design

A total of 801 US college students were recruited via Prolific.^4^ We targeted US American students using Prolific’s filters. We restricted the location to the US using ‘standard sample’, we set it to ‘Nationality: United States’, ‘Country of birth: United States’, ‘Place of most time spent before turning 18: United States’, ‘Student status: Yes’, ‘Minimum Approval Rate: 95, Maximum Approval Rate: 100’, ‘Minimum Submissions: 100, Maximum Submissions: 10000’. We first pretested survey duration with 30 participants to test the time run estimate and adjusted pay based on the duration. The data of the 30 participants were not analysed other than to assess technical issues, survey completion duration and needed pay adjustments and were included in the final data analysis. Table 2 compares sample characteristics and recruitment methods between the present replication and the original study [3].

**Table 2.** Difference and similarities between the original study and the replication.

	Norton *et al*. [3]				replication
	Study 1a	Study 1b	Study 2	Study 4	
sample size	294	49	76	190	801
geographic origin	not provided	not provided	not provided	not provided	US American students
gender	not provided	24 males, 25 females	30 males, 44 females, 2 did not disclose	68 males, 122 females,	351 males, 420 females, 27 other, 3 did not disclose
median age (years)	not provided	not provided	not provided	not provided	29.00
mean age (years)	not provided	19.7	24.1	34.1	31.50
standard deviation age (years)	not provided	2.5	10.3	11.9	10.85
age range (years)	not provided	not provided	not provided	not provided	18−71
medium (location)	computer (online)	MIT campus	MIT campus	computer (online)	computer (online)
compensation	not provided	not provided	not provided	not provided	nominal payment
year	2007	2007	2007	2007	2024

### 2.3. Procedure

Participants completed an online survey, which consisted of a consent form and replications of Studies 1a and 1b, 2 and 4, followed by funnelling and demographic information sections. The display of the studies and the conditions within each study were randomized.

We ran the four studies together in a single data collection. Combining several studies from a single target article in a single data collection has previously been successfully tested in several replications and extensions conducted by our team (e.g. [44–48]) and is especially powerful in addressing concerns about the target sample (naivety, attentiveness, etc.) when some studies replicate successfully, whereas others do not, as well as in the potential in drawing inferences about the links between the different studies and consistency in participants’ responding to similar decision-making paradigms. Unless explicitly noted, our measures are identical to those employed in Norton *et al*. [3].

### 2.4. Study 1a: Replication

Following the methods in Norton *et al*.’s [3] Study 1a, participants were asked to indicate which of two individuals about whom they know two different numbers of traits they think they would like more. More specifically, they were asked about a person about whom they knew 1 versus 2 traits, 2 versus 4 traits, 3 versus 6 traits, 4 versus 8 traits or 5 versus 10 traits. The question read, ‘Whom do you think you would like more, someone about whom you knew **X trait(s)** or someone about whom you knew **Y traits**?' with a binary choice between the two.

### 2.5. Study 1b: Replication

Participants indicated a choice between two options: ‘When you meet an individual for the first time, you tend to like that person more when…’ with the choice between ‘I know more about that person’ and ‘I know less about that person’. This served as the direct replication of Study 1b.

### 2.6. Study 2: Replication

Following Norton *et al*.’s [3] Study 2, participants were presented with a randomly selected set of traits taken from a previous study. Participants were randomly assigned to one of the four conditions varying in the number of the presented traits (4 versus 6 versus 8 versus 10 traits). These traits were randomly selected from a list of 28 traits generated by Norton *et al*. ([3, p. 99], footnote 3): *ambitious, boring, bright, critical, cultured, deliberate, dependable, emotional, enthusiastic, idealistic, imaginative, impulsive, individualistic, industrious, intelligent, level-headed, methodical, observant, open-minded, opinionated, polite, reliable, resourceful, self-disciplined, sensitive, stubborn, studious* and *talkative*.

Participants rated how much they would like an individual who possessed these traits—‘How much do you think you would like a person with the listed traits?’ (1 = *Wouldn’t like at all*; 10 = *Would like very much*).

### 2.7. Study 2: Extension and a conceptual replication of Study 3 in Norton *et al*. (2007)

#### 2.7.1. Curiosity (Extension)

As an extension to Study 2, after completing the procedure detailed above, participants also rated how curious they would be towards a person who possessed these traits—‘How curious would you be about a person with the listed traits?’ (1 = *Not at all curious*; 10 = *Extremely curious*).

#### 2.7.2. Similarity (Conceptual replication)

Participants rated how similar they perceive themselves to be to a person with these traits—‘How similar is the person with the listed traits to you?’ (1 = *Not at all*; 10 = *Extremely similar*).

### 2.8. Study 4: Conceptual replication/extension

Participants saw 10 randomly selected traits out of the list of 28 traits taken from Norton *et al*. [3] detailed above. These 10 traits were shown on different pages. Participants were asked to rate whether or not each of the 10 traits described themselves using a binary yes/no measure—‘Would you say that this trait describes you?’ (1 = *Yes*, 0 = *No*). Once all 10 traits were shown, participants were asked whether they would like a person who possessed these traits using a binary yes/no measure—‘Would you like a person who has the above 10 traits?’ (1 = *Yes*, 0 = *No*).

In addition, as an extension, we introduced continuous measurements of perceived similarity and liking of the target person after the first, fifth and tenth traits. The questions read: (i) Similarity—‘So far, how dissimilar/similar do you think the person is to you? (1 = *Extremely dissimilar*; 10 = *Extremely similar*)’ and (ii) Liking—‘So far, how much do you like the person? (1 = *Do not like the person at all*, 10 = *Like the person very much*)’, respectively, for perceived similarity and liking.

### 2.9. Data analysis strategy

#### 2.9.1. Replication hypotheses: H1a–H4-2

Evaluations of replication were made based on the LeBel *et al*. [22] criteria. Following Norton *et al*. [3], we conducted a chi-square test to test H1a that people prefer a person whom they know more about over a person whom they know less about.

To test H1b, we ran a chi-square test, examining the correlation between the number of traits described for a target person and the liking of that person.

To test H21, we computed a correlation between the number of pieces of information about a person and the degree of liking.

To test H2-2, we conducted a 1 × 4 (the number of pieces of information: 4 versus 6 versus 8 versus 10) between-subjects design ANOVA on liking and follow-up the analysis with post hoc pairwise comparisons with *p*-values adjusted by the Holm method. To meet the hypotheses, the three comparisons (4 versus 6, 6 versus 8 and 8 versus 10) should all have a signal such that participants like the person less when they receive more pieces of information about the person.

To test H3, we conducted an exploratory mediation model in which perceived similarity mediates the relationship between the number of pieces of information about a person and how much participants like the person. While Norton *et al*. [3] tested the mediating effect with the method proposed by Baron & Kenny [49], we tested the mediation effect with adjusted bootstrap percentile (BCa) methods.

To test H4-1 and H4-2, we first created quasi-experimental conditions based on whether participants find the first presented trait of a person similar or dissimilar to themselves. We then conducted a Welch’s *t*‐test to examine whether those in the dissimilar condition rate the subsequently presented traits as being more dissimilar than those in the similar condition (H4-1). To test H4-2, we conducted the same analysis on liking.

#### 2.9.2. Extensions hypotheses: H5–H9

As an extension, using data from the replication of Study 2, we first computed bivariate correlations among the number of pieces of information available, curiosity and liking. We had the following two extension hypotheses; H5: The number of pieces of information someone knows about a person negatively correlates with curiosity towards them; H6: Curiosity is positively correlated with degree of liking. We pre-registered that if H5 and H6 were supported, we would test a mediation model in which the number of pieces of available information about a person has indirect effects via perceived similarity and curiosity. We expected that curiosity then has an indirect effect between knowledge and liking, partly explaining the *less is more* effect (H7).

To better elucidate the cascading effect of the instance of dissimilarity, using data from the replication of Study 4, we examined how perceived similarity and liking change over time (i.e. when presented with the first, fifth and tenth trait). We expected that people in the dissimilar condition (i.e. those who perceive the first presented trait as evidence of dissimilarity) would like the target less and less as they received more pieces of information about the target person (H8). Similarly, we predicted that they would perceive the target to be more and more dissimilar to them as they received more pieces of information about the person (H9). To test these hypotheses, we focused on participants in the dissimilar condition and built a linear mixed model in which liking or perceived similarity were regressed on the two dummy-coded variables of the number of presented traits (5 versus 1 and 10 versus 5). Given participants rated liking and perceived similarity three times, we treated participants as a random effect in the model and let the intercept vary.

#### 2.9.3. Order effects (exploratory)

One deviation from the target article was that all participants completed all studies in a random order. We considered this to be a stronger design with many advantages, yet one disadvantage is that answers to one scenario may bias participants’ answers to the following scenarios.

We thus ran exploratory analyses focusing on the participants that completed that study first and reported the differences between the two, examining whether the confidence intervals of the effect sizes overlap. To compensate for multiple comparisons and increased likelihood of capitalizing on chance, we set the α for the additional analyses to a stricter 0.005.

#### 2.9.4. Outliers and exclusions

We did not classify outliers in this study. All data from participants who successfully completed the survey were included.

#### 2.9.5. Replication closeness evaluation

We provide details on the classification of the replications using the criteria by LeBel *et al*. [50] in Table 3. We summarized the replication as a close replication.

**Table 3.** Classification of the replications (Studies 1a, 1b, 2 and 4), based on LeBel *et al*. [50].

design facet	replication	details of deviation	reason for deviation
effect/hypothesis	same		
Studies 1a and 1b	same		
Study 2	same+	we retained Norton *et al*.’s [3] hypotheses but also included additional hypotheses (H5−7)	this allowed us to explore curiosity as a potential pathway between knowledge and degree of liking
Study 4	same+	we retained Norton *et al*.’s [3] hypotheses but also included additional hypotheses H8 and H9	this allowed us to further elucidate the effect of dissimilarity cascades and their influence on liking
IV construct	same		
DV construct	same		
Studies 1a and 1b	same		
Study 2	same+	we retained constructs from Norton *et al*.’s [3] original study but also measured perceived similarity to target	this allowed us to conceptually replicate the findings from Study 3 by Norton *et al*. [3]
Study 4	same		
IV operationalization	same		
DV operationalization	same		
Studies 1a and 1b	same		
Study 2	same		
Study 4	same+	we retained measures by Norton *et al*. [3] but also included continuous measures of perceived liking and similarity at traits 1, 5 and 10	this increased sensitivity of the measures of liking and similarity and allowed us to explore their change over time as more traits are known
population (e.g. age)	similar		
Study 1 a	different	the target article’s study recruited participants via an online dating website. The replication used an online US undergraduate student sample recruited via Prolific	conducting an online study ensured we had sufficient power at a reasonable cost
Study 1b	similar	the target article’s study recruited MIT undergraduates. The replication used an online US undergraduate student sample recruited via Prolific	
Study 2	similar	the target article’s study recruited individuals from MIT campus. The replication used an online US undergraduate student sample recruited via Prolific	
Study 4	similar	he target article’s study recruited MIT and Yale students. The replication used an online US undergraduate student sample recruited via Prolific	
IV stimuli	same		
DV stimuli	same		
Studies 1a and 1b	same		
Study 2	same+	we retained DV stimuli from Norton *et al*. [3] but also included a measure of perceived similarity to the target	this allowed us to conceptually replicate Study 3
Study 4	same+	we retained DV stimuli from Norton *et al*. [3] but also included continuous measures of perceived liking and similarity at trait 1, 5 and 10	this allowed us to explore the influence of dissimilarity cascades on degree of liking as more information is known
procedural details	similar	the four studies were combined in the replication	conducting a single study ensured we had sufficient power at a reasonable cost
Studies 1a and 1b	similar	see above	see above
Study 2	similar	see above	see above
Study 4	similar	we included a continuous measure of perceived similarity and a continuous measure of degree of liking at traits 1, 5 and 10. As such, we treat Study 4 as a conceptual replication	inclusion of these questions increases sensitivity to the perceived similarity and degree of liking measures above those used in the original study. Measuring these at three time points allowed us to examine their change as more information about the target is known
physical settings	different		
Study 1a	same		
Study 1b	different	original article recruited participants by approaching them in the campus student centre. Replication was an online survey	conducting a single online study ensured we had sufficient power at a reasonable cost
Study 2	different	original article recruited participants by approaching them on MIT campus or as part of class exercise. Replication was an online survey	conducting a single online study ensured we had sufficient power at a reasonable cost
Study 4	different	original article recruited participants as part of class exercise or as part of a web-based survey for a series of unrelated experiments. Replication was an online survey	conducting a single online study ensured we had sufficient power at a reasonable cost
contextual variables	different	different time and context	
replication classification	close replication		

#### 2.9.6. Missing data

One participant did not answer one question. We retained their response for analysis. All other participants answered all questions.

#### 2.9.7. Deviation from the Stage 1 pre-registration plan

We report no major deviations from the pre-registered protocols for the data collection and analysis. During analysis after data collection, we identified and corrected an oversight in the code for H4-2 and also optimized the code for better reproducibility, reporting and plotting. The updated code and associated outputs are provided on the Open Science Framework (OSF).

## 3. Results

### 3.1. Replication of Study 1a: H1a and H1b

We first conducted a chi-square test to test H1a. We found that people indicated that they preferred a person who they know more about (*n* = 643) over one they know less about (*n* = 158; χ(1) = 293.7, *p* < 0.001, *h* = 0.65, 95% CI [0.55, 0.75]). We found the same for each comparison (1 versus 2 traits, 2 versus 4 traits, 3 versus 6 traits, 4 versus 8 traits and 5 versus 10 traits; χ_s_ > 25.6 , *p_s _*< .001).

To test H1b, we conducted a chi-square test and examined whether participants believe that more information leads to more liking. We found that the more people thought they would like a person more, the more they knew more about them (*n* = 648) than people who thought they would like a person more the less they knew about them (*n* = 153; χ(1) = 305.9, *p* < .001, *h* = 0.67, 95% CI [0.57, 0.76]).

We concluded support for H1a and H1b and a successful replication of Studies 1a and 1b.

### 3.2. Replication of Study 2: Hypotheses H2-1 and H2-2

We found no support for an association between the number of pieces of information about a person and the degree of liking (H2-1; *r* (799) = −0.027, 95% CI [−0.097, 0.042], *p* = .325).

We further conducted supplementary analyses with a 1 × 4 (the number of presented traits: 4 versus 6 versus 8 versus 10) between-subjects ANOVA on liking. We found an indication for differences between the conditions varying the number of presented traits (*F*3,797 = 3.17, *p* = .024, η_p_^2^ = 0.01). Following the pre-registration, we then ran post hoc pairwise comparisons with the Holm method adjustment and only found support for differences in one comparison: 4 traits versus 8 traits (*p*_Holm_ = .038), with higher liking for a target with 4 traits than towards the target with 8 traits. We found no support for other differences (*p_s _*> .050).

We therefore concluded failure to find support for H2-1 and H2-2, with no consistent evidence that people liked a person with fewer known traits more than one with more known traits.

### 3.3. Conceptual replication in Study 2 of target’s Study 3: Hypothesis 3 (exploratory)

We built a partial mediation model in which the number of the presented pieces of information about a target had an indirect effect on liking towards the target via perceived similarity to them. We found no support for the positive relationship between the number of presented traits and similarity (*β* = 0.02, 95% CI [−0.05, 0.08], *p* = .588), support for the path between similarity and liking (*β* = 0.73, 95% CI [0.68, 0.77] *p* < .001), and no support for the indirect effect of the number of presented traits on liking via perceived similarity (*β* = 0.01, 95% CI [−0.003, 0.006], *p* = .588).

We thus concluded no support for H3 and a failure to replicate the original mediation effect.

### 3.4. Conceptual replication of Study 4: Hypotheses 4-1 and H4-2

**Figure 1.**
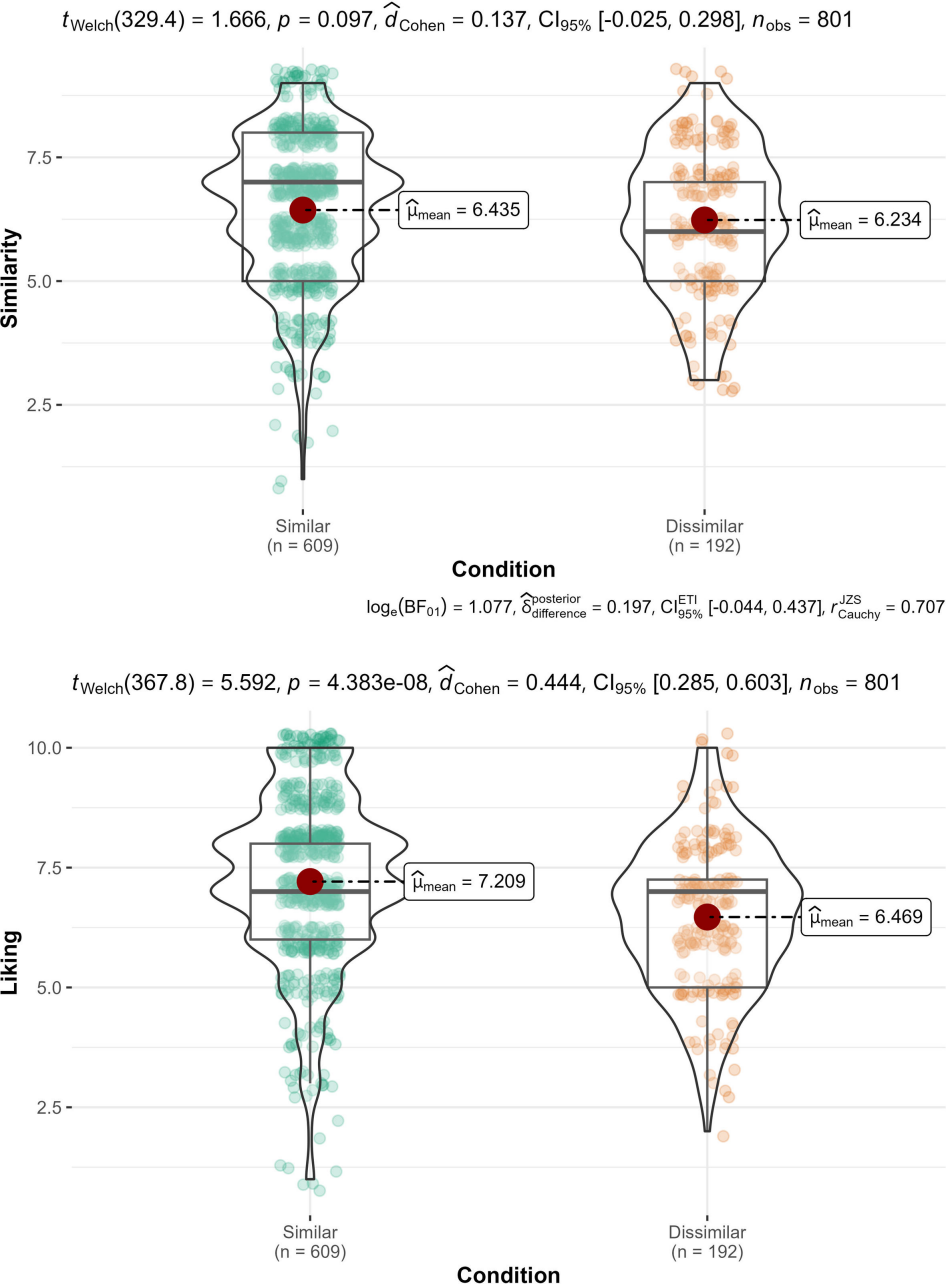
Study 2: Perceived similarity and liking by condition.

We first categorized participants into two groups based on whether they indicated that the first presented trait about a target person described themselves or not (similar versus dissimilar groups). To test H4-1, we conducted a Welch’s *t*‐test to examine whether people in the dissimilar condition (i.e. those who found the first presented trait *not* describing themselves) perceived the target person as more dissimilar to themselves overall compared to those in the similar condition (i.e. those who found the first presented trait to describe themselves). We found no evidence for differences in perceived similarity between the two conditions (H4-1; plotted in Figure 1; *t*(329.4) = 1.67, *p* = .097, *d* = 0.14, 95% CI [−0.03, 0.30]).

We conducted the same analysis with liking as the dependent variable and found evidence for those under initial impressions of dissimilarity liking the target less after all traits were shown (*t*(367.8) = 5.59, *p* < .001, *d* = 0.44, 95% CI [0.28, 0.61]), indicating support for H4-2.

### 3.5. Extensions of Study 2: H5-H7

We computed correlations between curiosity, the number of pieces of information, and liking. We failed to find support for the association between curiosity and the number of pieces of information (H5; *r*(799) = −.01, 95% CI [−0.08, 0.06], *p* > .999) and therefore did not proceed to conduct the mediation model regarding curiosity (H7). We found support for a positive correlation between curiosity and liking (H6; *r*(799) = .66, 95% CI [0.62, 0.70], *p* < .001).

### 3.6. Extensions of Study 4: H8–H9

**Figure 2.**
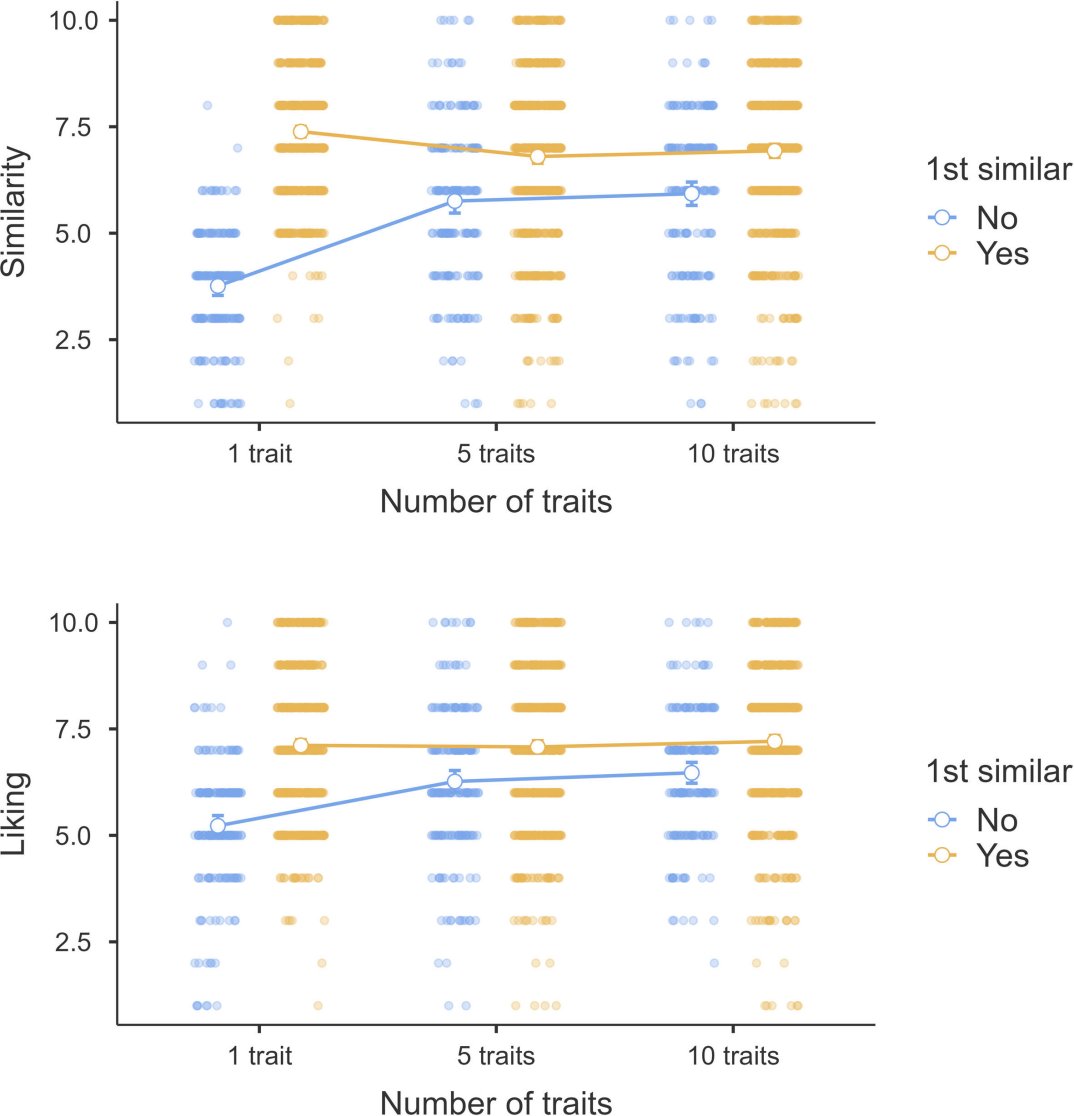
Study 4 cascading effect: perceived similarity (top) and liking (bottom) by condition.

Following our pre-registration plan, we dummy-coded the number of the presented traits (5 versus 1, 10 versus 5) and tested whether perceived similarity and liking declined as participants received more information about the target, focusing only on participants who rated the first trait as dissimilar to themselves (*n*_obs_ = 576). We plotted the interaction in Figure 2.

We then built a linear mixed model in which perceived similarity and liking were each regressed on the two dummy-coded variables (fixed effects) and let the intercept vary for each participant. We found support for an *increase* in perceived similarity between seeing 1 versus 5 traits (*β*(382) = 1.99, 95% CIs [1.69, 2.30], *t* = 12.71, *p* < .001). Between 5 versus 10 traits, we found no indication for a change in perceived similarity (*β*(1958) = 0.17, 95% CI [−0.14, 0.48], *t* = 1.10, *p* = .027).

We observed a similar trend for liking, with an increase between traits 1 and 5 (*β*(382) = 1.04, 95% CI [0.78, 1.31], *t* = 7.66, *p* < .001) but with no indication for a change between traits 5 and 10 (*β*(382) = 0.20, 95% CI [−0.06, 0.47], *t* = 1.49, *p* = .14).

We therefore conclude that our findings challenge H8 and H9.

### 3.7. Order effects

We found no indication of order effects and reported all order related findings in the electronic supplementary materials.

## 4. Discussion

We conducted a series of direct and conceptual replications of the target article’s original Studies 1–4. Taken together, our findings show little support for the *less is more* effect proposed by Norton and colleagues.

### 4.1. Replications

We were able to replicate the original finding from Studies 1a and 1b that people expected they would like a target more when they knew more about them. In our replication of Study 1a, we found that participants were more likely to prefer an individual with a higher number of known traits over one with fewer known traits. In our replication of Study 1b, people predicted greater liking towards others they knew more about.

Our attempts to replicate the effects of actual knowledge on liking were less successful. In our replication of Study 2, we failed to find support for a linear relationship between the number of traits presented and the degree of liking towards the target. While additional analyses did not find support for variations in liking based on the number of traits presented, we note that a pairwise comparison revealed greater liking towards a target with four known traits compared to one with eight known traits. This finding aligns with the expected direction of the *less is more* effect, though it is unclear why this effect emerged for this contrast only. Overall, our results do not consistently replicate previous findings from Norton *et al*. [3]. Instead, in line with prior research [21], our results challenge the *less is more* effect and suggest no significant impact of knowledge on liking.

This highlights a potential gap between individuals’ expected versus observed attitudes during impression formation, while also underlining the theoretical disparity between ‘knowing’ and ‘liking’. Our findings, suggesting that more information does not lead to reductions in perceived liking, indicate that the process of acquiring knowledge may operate independently of the formation of affect-based attitudes. This notion aligns with prior literature emphasizing the importance of distinguishing between cognitive- versus affect-based attitudes in other contexts (e.g. Persuasion; 51). Distinguishing between cognitive-based attitudes such as perceived knowledge of an individual versus affect-based attitudes such as liking may offer a new perspective on understanding the precise roles of knowledge and similarity in attitude formation.

The person positivity bias [12] and false consensus effect [16] may contribute to this disconnect between expected versus observed associations between knowledge and liking, where unrealistic expectations are fostered through incorrect assumptions of similarity and, therefore, liking. Given these initial assumptions of similarity between oneself and unfamiliar targets, it is reasonable to assume we would anticipate any new information we learn about them to evidence further similarity, explaining the false belief that knowing more about an individual fosters further positive affect-based attitudes (i.e. liking). We are unlikely to be aware of the influence of this bias on initial impression formation; social biases can occur unconsciously (e.g. the halo effect [52]), and there is a tendency to incorrectly attribute perceptions of (dis)like towards an attitude target to irrelevant factors [53]. In the context of knowledge and liking, this lack of awareness may lead individuals to limit their opportunity for personal growth and positive social interactions. For instance, they may perpetuate social division by dismissing opportunities to learn about new cultures if those experiences do not lead to the positive emotional outcomes they expect. They may even attempt to rationalize or minimize negative feelings that arise from instances of cognitive dissonance caused by inconsistencies between the anticipated versus (lack of) observed positive affect from learning about others, leading to avoidance rather than attempting to integrate this new knowledge to generate more accurate judgements towards them.

In Study 2, we attempted to replicate Norton *et al*.’s [3] findings for a mediating role of dissimilarity in the relationship between knowledge and liking (original Study 3). Specifically, the authors predicted (and found) that the *less is more* effect is explained by increasing perceptions of dissimilarity during learning; as we learn more about an individual, our overly optimistic assumptions of similarity are challenged, and the degree of liking towards them subsequently diminishes. Our findings failed to replicate the original results. While similarity and liking were positively related (aligning with prior evidence for the *similarity-attraction paradigm*; see [17]), the number of known traits had no effect on perceived similarity. Consequently, our findings showed no evidence that similarity mediates the knowledge-liking relationship, failing to support the notion that learning breeds contempt by disputing assumptions of similarity and further supporting a distinction between cognitive-based (i.e., perceived similarity) versus affect-based (i.e., perceived liking) attitudes (51) in impression formation contexts.

Finally, Norton and colleagues [3] proposed a mechanism of *dissimilarity cascades*, whereby initial impressions of dissimilarity to the target result in subsequent information being interpreted as further evidence of dissimilarity. Our attempts to replicate this effect showed mixed success. While Norton and colleagues found that initial impressions of dissimilarity predicted subsequent perceptions of (dis)similarity and liking, our findings supported this effect for subsequent perceptions of liking only, with lower liking for those initially perceived as more dissimilar.

Since we found no support for the effect on perceived similarity, we therefore concluded that there was no support for dissimilarity cascades in impression formation. Our findings align more closely with the information integration hypothesis [21], which suggests that people process information about others in an unbiased manner to form an overall final impression of the target. This does not, however, explain why those under initial impressions of dissimilarity to the target showed less liking towards them after all traits were presented, though it further strengthens claims for the previously mentioned independence of cognitive- versus affect-based attitudes.

### 4.2. Extensions

We extended Norton *et al*.’s [3] research by introducing a new variable, curiosity. Norton and colleagues originally asserted that the *less is more* effect is partly driven by the *lure of ambiguity*; over-estimations of similarity to others in the absence of knowledge ([13–15]; see also [16]) are challenged as more information is learnt.

Asserting a novel account for this mechanism, we predicted that curiosity may also mediate the negative relationship between knowledge and liking. Specifically, knowing only a few traits about an individual may trigger feelings of curiosity, which increases attraction towards an individual due to the desire to learn more about them [36]. As the amount of knowledge increases, this curiosity diminishes, along with the associated attraction towards the target.

We failed to support this prediction, however, owing in part to the unsuccessful replication of the *less is more* effect. While curiosity was positively correlated with degree of liking (potentially supporting the driving effect of curiosity on attraction [36]), the number of known traits did not influence curiosity towards the target. Since the effect of knowledge on curiosity did not emerge, we were thus unable to test the proposed mediation model. Consequently, we were unable to yield evidence for the predicted role of curiosity in impression formation, though we do not challenge the possibility of this effect emerging outside of the current study context.

Finally, we adjusted the original design of Norton *et al*.’s [3] Study 4 to better elucidate the dissimilarity cascade effect by examining how perceived similarity and liking changed over time (i.e. when presented with the first trait, fifth and tenth traits) in those who initially perceived the target as dissimilar to themselves (i.e. rated the first presented trait as dissimilar to themselves). Our findings yielded mixed evidence for the roles of knowledge and similarity in impression formation. Directly challenging the notion of dissimilarity cascades, our findings showed that perceived similarity *increased* from seeing the first trait to seeing the fifth. As such, contrary to claims by Norton and colleagues, we found no indication that initial impressions of dissimilarity cause subsequent evidence to be interpreted as further evidence of dissimilarity. Again, these findings align with the information integration hypothesis from Ullrich *et al*. [21], suggesting the way information about others is processed is not biased by preceding information. Beyond the fifth trait, however, seeing more traits did not influence similarity ratings, indicating no further effects of knowledge on similarity perceptions.

Interestingly, we found an identical effect for liking, whereby liking increased between traits 1 and 5 but plateaued from the fifth trait onwards. This suggests the relationship between knowledge and attraction may be more complex than previous research has suggested. Specifically, under initial impressions of dissimilarity, learning more about an individual may facilitate perceptions of similarity and liking towards the target. When the target is perceived to be similar, however, this effect plateaus; uncovering additional traits about the individual has no further influence on liking or similarity. Supporting this notion, differences between initial similarity conditions for both liking and similarity ratings were significant only between the first and fifth traits, becoming non-significant between the fifth and tenth. As such, our findings indicate that the *more is more* effect may emerge only under initial impressions of target dissimilarity, and diminish once feelings of dissimilarity are overcome.

Taken together, these findings challenge the *less is more* effect and offer a more nuanced account of the relationship between knowledge of a target and liking than the information integration hypothesis [21] proposes.

### 4.3. Implications, limitations, and directions for future research

We conducted our replications using a unified data collection strategy, running all studies in the same data collection. This method afforded several benefits. The unified design showed that the failure to support some studies (Studies 2 and 4) is likely not due to an inattentive or unique sample, given that with the very same participants we were able to successfully replicate findings for other studies (Studies 1a and 1b). In addition, the unified design showed that the same participants who expected a positive association between knowing a person and liking did not exhibit such a relationship. Yet the unified design also introduced the possibility of order effects. We anticipated this in advance and pre-registered tests for order effects yet found no indication for differences in effect sizes or order effects. Therefore, we do not believe that the unified design is the reason for our unsuccessful replication of the *less is more* effect.

Our sample was of US college students recruited online, with the aim of remaining close to the samples used in the original studies (students from Yale and MIT). We acknowledge the possibility that effects may be different in other samples, such as that effects would be more consistent with the target’s findings if run with student samples from the same universities. However, we consider this unlikely, and—if true—it would cast doubt on the generalizability and importance of the phenomenon. It is also possible that both the original and the replication effects would vary if studied in non-student samples or with samples outside of the USA. Future research may thus explore whether perceptions of knowledge and (dis)similarity in impression formation vary by sample and/or culture.

Additionally, we did not pursue a direct replication of Norton *et al*.’s [3] Studies 3 and 5. These studies tested the effects of familiarity and similarity on liking using online dating platform users (Study 3) and in real-world dating contexts (Study 5). It is possible that our findings, conducted online and using imagined scenarios, would be different if tested in real-world contexts [5]. Future research could thus extend our current replication efforts by conducting a replication in real-world settings, though we believe that our failed replications of well-controlled and less-noisy lab experiments make an investment in pursuing such a study risky.

While we were unable to test the mediating role of curiosity in the *less is more* effect since the effect did not emerge, we did find some preliminary evidence for the effects of curiosity with liking, in line with previous research [36]. The association between curiosity and liking, partnered with the lack of association between curiosity and the number of traits shown, may suggest that curiosity in the context of liking may not be primarily focused on filling a quantifiable information gap. Instead, it may be related to the relevance or quality of the information known, or other unknown factors that may impact on the subjective experience of curiosity and its affective outcomes. A potential suggestion could be to explore both state and trait curiosity within future research and focus on the particular motivations underlying curiosity to expand upon our current understandings of impression formation.

Our findings from Study 4 provided mixed evidence for the role of initial similarity impressions in subsequent attitude formation. While liking and similarity perceptions increased in the initial dissimilarity condition as more traits were presented, those who initially rated the target as dissimilar to themselves still showed less liking towards the target compared to those in the initial similarity condition. These differences could not be explained by perceptions of similarity, since similarity scores did not significantly vary between conditions.

We did not investigate how perceived liking and similarity changed under initial impressions of similarity to the target. Therefore, the trend observed in Study 4, whereby liking and similarity increased between the first and fifth traits under initial impressions of dissimilarity, may also emerge under initial impressions of similarity to the target. The operationalization of these factors in the original study and current replication does not lend itself to studying this effect due to potential ceiling effects. Future research may aim to overcome these issues by asking participants to rate changes in perceived liking and similarity over time, rather than merely rating their current attitudes towards the target.

A more general limitation in research exploring initial impression formation (e.g. [3,10]) is regarding the conflation between familiarity and knowledge of an individual. As Reis *et al*. [5] note, familiarity with others requires interpersonal interaction, not just knowledge of facts. The target article and our replication do not fully capture familiarity but rather its superficial subfacet of knowledge and regard somewhat ambiguous information with a brief description of traits. Future research looking into this relationship may seek to improve on the target’s methods to allow study of more in-depth knowledge and interactions that lead to familiarity to see whether those may impact liking and/or similarity perceptions differently.

Finally, our results further the growing recognition for the importance of replicating social psychological research [54,55]. To tackle inconsistencies in the literature (including our own), we echo Norton *et al*.’s [10,19] calls to explore potential moderating factors of the knowledge-liking relationship. For example, conducting meta-analytical investigations of extant studies on the *less is more* effect may yield insights into the potential moderating effects during real-world impression formation (e.g. target gender [10]), or methodological disparities in prior research such as operationalization of knowledge (e.g. manipulating the number of traits of a hypothetical target shown [3,21] versus self-reported measures of interaction-generated knowledge [5], informing future efforts to test this effect.

## 5. Conclusion

We successfully replicated the expectations that knowing a person better would be associated with liking them more, yet failed to replicate the *less is more* effect proposed by Norton *et al*. [3] showing that manipulating knowledge about people impacts liking. Our findings point to a more complex role of knowledge in attraction, suggesting that learning more about an individual may increase perceived similarity and liking towards them (the *more is more effect*), yet more only when initial evidence indicates dissimilarity. We conclude that learning more about others may function to overcome early negative impressions but has little influence once a positive impression is achieved.

## Data Availability

Project finished. We provided all materials, data, and code on: [56]. Supplementary material is available online [57].

## Footnotes

^1^
We use the word acquaintance to refer to someone whom an individual has previously encountered, butremains largely unfamiliar; for example, sight-based recognition from an initial interaction.

^2^
We chose not to include Study 5 by Norton *et al*. [3] in our replication. Study 5 explored whether findings from Studies 1–4 would be replicated in real-life settings, using individuals from a real-world dating platform who had either recently been on a first date, or were going on a first date in the near future. Findings replicated effects reported in Studies 1–4. We felt that we should first focus on Studies 1–4 and once we are able to establish the replicability and robustness of those findings, inspire the more costly and ambitious follow-up of Study 5 in real-life setting.

^3^
Although the power for this study to detect each hypothesized effect is at least 80%, the power of this study to detect all of these effects simultaneously may be lower.

^4^
Our planned sample size was 800 participants. In some instances, Prolific recruits participants beyond the specified sample size. This is due to the platform sometimes incorrectly classifying valid completed responses as ‘timed out’ or ‘returned’. We followed our pre-registration plan to not exclude any complete valid responses from our dataset, including any additional completed responses obtained from Prolific.

## Authors' Notes

Figures 1 and 2 were created using ggstatsplot [58] and JAMOVI [59].
